# Cryo-EM structures of human TMEM120A and TMEM120B

**DOI:** 10.1038/s41421-021-00319-5

**Published:** 2021-08-31

**Authors:** Meng Ke, Yue Yu, Changjian Zhao, Shirong Lai, Qiang Su, Weidan Yuan, Lina Yang, Dong Deng, Kun Wu, Weizheng Zeng, Jia Geng, Jianping Wu, Zhen Yan

**Affiliations:** 1 Key Laboratory of Structural Biology of Zhejiang Province, School of Life Sciences, Westlake University, Hangzhou, Zhejiang China; 2 Westlake Laboratory of Life Sciences and Biomedicine, Hangzhou, Zhejiang China; 3 Institute of Biology, Westlake Institute for Advanced Study, Hangzhou, Zhejiang China; 4 Department of Laboratory Medicine, State Key Laboratory of Biotherapy and Cancer Center, West China Hospital, Sichuan University and Collaborative Innovation Center, Chengdu, Sichuan China; 5 Key Laboratory of Birth Defects and Related Disease of Women and Children of MOE, West China Second Hospital, Sichuan University, Chengdu, Sichuan China; 6 Emergency Medicine Clinical Research Center, Beijing Key Laboratory of Cardiopulmonary Cerebral Resuscitation, Medical Research Center, Beijing Chao-Yang Hospital, Capital Medical University, Beijing, China

**Keywords:** Cryoelectron microscopy, Ion channel signalling

Dear Editor,

Pain is a protective signal of impending danger, which is essential for our interaction with the environment^1^. The identification of related mechanosensing ion channel (MSC) is fundamentally important for understanding the mechanism of mechanical pain sensing and analgesic drug discovery. A recent study identified TMEM120A, also named TACAN, as an MSC that contributes to mechanosensitive currents in nociceptors^2^. TMEM120A shares no sequence homology with any reported MSCs or other channels. Despite sequence similarity, no mechanically evoked current was detected for heterologously expressed TMEM120B, a homolog of TMEM120A^2^. TMEM120A/B were originally identified as nuclear membrane localized protein that plays an important role in adipocyte differentiation^3,4^. A recent study shows that adipocyte-specific knockout of TMEM120A leads to latent lipodystrophy^5^. The molecular basis underlying the physiological functions of TMEM120A is elusive.

We carried out a comparative structural investigation between TMEM120A and TMEM120B, attempting to uncover their potential difference in mechanosensing property. Here, we present the cryo-electron microscopy (cryo-EM) structures of both human TMEM120A and TMEM120B at overall resolutions of 3.7 Å and 4.0 Å, respectively. Details about the sample preparation, data collection, and processing can be found in Supplementary figures and Methods (Supplementary Figs. S1–S3). The cryo-EM maps of TMEM120A and TMEM120B reveal a shared homo-dimeric assembly, which is about 80 Å in height and 85 Å in width (Fig. 1a). The two structures are nearly identical with a root-mean-square distance (r.m.s.d.) of only 0.82 Å over 293 Cα atoms when superimposed with each other (Fig. 1b).

**Fig. 1:**
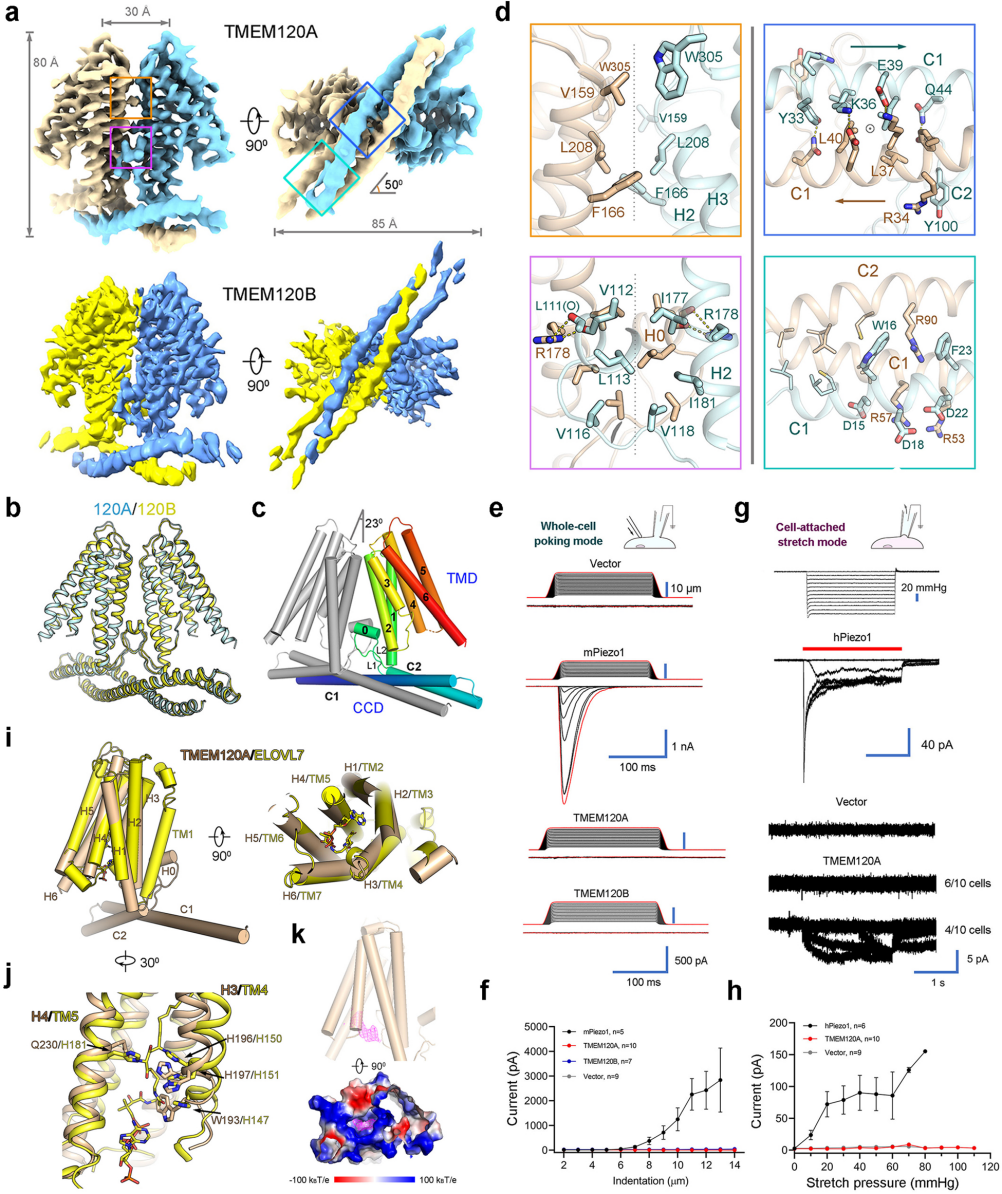
Cryo-EM structures and electrophysiological characterizations of human TMEM120A and TMEM120B. **a** Cryo-EM maps of human TMEM120A and TMEM120B shown in two perpendicular views. Densities for the detergent micelle were omitted for visual clarity. The maps were contoured at level 0.7 and 0.6 for TMEM120A and TMEM120B in ChimeraX^9^, respectively. The dimer interfaces that are highlighted in the boxes are illustrated in detail in **d** with corresponding color borders. **b** TMEM120A and TMEM120B share nearly identical overall structure. TMEM120A and TMEM120B are colored cyan and yellow, respectively. **c** Structural topology of TMEM120A. One protomer is colored gray and the other is colored rainbow with the N- and C-terminus in blue and red, respectively. TMD, transmembrane domain; CCD, coiled coil domain. **d** Close-up views of the dimeric interfaces of TMD (left) and CCD (right). The TMD interfaces are along the C2 symmetry axis, which is indicated by the gray dashed lines. The C2 symmetry axis in the CCD is indicated by the circle in the upper panel. **e** Representative traces of poking-evoked whole-cell currents from HEK293T-P1KO cells transfected with mPiezo1 and TMEM120A, or from HEK293T cells transfected with TMEM120B and vector. **f** Statistic plot of the poking-evoked currents along the increase of indentation depth. **g** Representative traces of stretch-induced currents in a cell-attached mode from HEK293T-P1KO cells transfected with hPiezo1, TMEM120A, and vector. The stretch durations are indicated by red bars. **h** Statistic plot of the stretch-induced currents along the increase of negative pressure. **i** Structural comparison between TMEM120A and ELOVL7 elongase (PDB: 6Y7F), shown as cylindrical helices in two perpendicular views. The 3-keto acyl-CoA bound in ELOVL7 is shown as sticks. **j** Close-up view of the superimposition near the HxxHH motif in the catalytic site of ELOVL7. **k** A stretch of electron density is identified in the intracellular pocket of TMEM120A, which is surrounded by positively charged residues. The density is contoured at 4σ. Structure figures were prepared in PyMOL^10^.

We select TMEM120A for detailed analysis unless otherwise stated. Both the N-terminus and the C-terminus of each protomer are located on the intracellular side. The transmembrane domain (TMD) of each protomer contains six transmembrane helices, designated H1—H6, enclosing a large solvent-accessible cytosol-facing cavity. The N-terminal cytosolic regions from both protomers together form the coiled coil domain (CCD), with each protomer contributing two horizontal helices C1 and C2 (Fig. 1c; Supplementary Fig. S4a). In addition, a short amphipathic helix H0 parallel to the inner membrane was identified between C2 and H1, flanked by two intracellular linkers L1 and L2 (Fig. 1c). When viewed from the cytosolic side, long axes of CCD and TMD form an angle of approximately 50° (Fig. 1a).

The dimeric assembly is stabilized by extensive interactions in both CCD and TMD (Fig. 1d). The C1 helices from the two protomers form an anti-parallel coiled coil through multiple types of interactions, including hydrogen bonds, cation—π interactions, and van der Waals contacts. At the two ends of the coiled coil, the short C2 helix jointly forms two 3-helix bundles (Fig. 1d). The dimer interface in TMD, which is along the C2 symmetry axis, is mostly mediated by van der Waals contacts. Near the extracellular side, Val159 and Phe166 on H2, Leu208 on H3, and Trp305 on H6 from the two protomers contribute to the interface interaction. On the interface near the intracellular side, three layers of hydrophobic residues from L2, H0, and H2 interlock with each other (Fig. 1d). In addition, Arg178 on H2 of one protomer forms hydrogen bonds with the carbonyl oxygens of Leu111 and Val112 on H0′ of the other protomer. These specific interactions stabilize H0, which in turn buttress dimerization (Fig. 1d).

Most of the residues in the dimer interface are highly conserved between TMEM120A and TMEM120B (Supplementary Fig. S5). The CCD is the less conserved region, but the overall structures of CCD between the two proteins are the same. From the structural perspective, there is hardly any hint for the difference between TMEM120A and TMEM120B in sensing mechanical force.

We also examined the electrophysiological property of TMEM120A in both the bilayer system and heterologous expression system. We purified the full-length human TMEM120A and TMEM120B and reconstituted them into a planar lipid bilayer for conducting the current recording. A discrete current increase, measured at the voltage of +10 mV, was observed after reconstitution of purified TMEM120A (Supplementary Fig. S6a). TMEM120A showed an almost linear current–voltage (*I*–*V*) relationship from –50 mV to +50 mV, which is consistent with the reported electrophysiological behavior in bilayer^2^ (Supplementary Fig. S6b, c). We also examined TMEM120B using the same system, and the current mediated by TMEM120B was slightly smaller than that by TMEM120A at the same voltage level (Supplementary Fig. S6d, e).

We tested the mechanosensing property of TMEM120A by two mechanical force modes in a heterologous expression system, in which the plasmids of TMEM120A were transiently transfected into HEK293T or HEK293T-P1KO cells. Under the whole-cell poking mode, we applied steps of mechanical stimuli on the measured cells by a fire-polished glass pipette with 200 ms duration. Cells transfected with mouse Piezo1 showed significantly increased mechanically evoked currents compared with the empty vector-transfected cells (Fig. 1e). By contrast, no significant difference was detected between cells transfected with TEME120A/B and the empty vector-transfected cells (Fig. 1e, f). We also measured the stretch-induced currents by applying negative pressures under the cell-attached stretch mode. The positive control cells transfected with human Piezo1 mediated robust stretch-induced currents and the amplitude of the currents was improved with the increasing stretch pressure (Fig. 1g, h). Out of ten measured cells transfected with TMEM120A, six showed no obvious stretch-induced currents, similar to the negative control cells transfected with vector. The other four cells displayed currents in ~5 pA level, which is likely to be leaky currents for two reasons. First, the amplitude of the current is not proportional to the stretch pressures. Second, the currents exist even after the pressure is relieved. Therefore, based on our experimental results, TMEM120A could mediate conducting currents in a bilayer system, but does not behave as an MSC in heterologous system.

A search of TMEM120A protomer in the DALI^6^ server identified a structurally homologous protein ELOVL7^7^, an endoplasmic reticulum-embedded fatty acid elongase involved in the elongation of very long chain fatty acid. ELOVL7 catalyzes the condensation reaction step between an acyl-CoA and a malonyl-CoA to form a 3-keto acyl-CoA, followed by three other steps to yield a product acyl-CoA with two extra carbons. ELOVL7 contains seven TM helices, TM1—TM7. Structural comparison between TMEM120A and ELOVL7 reveals similar fold in the TMD. The H1—H6 of TEME120A can superimpose with TM2—TM7 of ELOVL7, with an r.m.s.d. of ~2.8 Å over 193 aligned Cα atoms (Fig. 1i). Notably, two histidine residues His150 and His151 of the critical HxxHH motif in the catalytic site of ELOVL7 are also conserved in TMEM120A (Fig. 1j). In addition, like ELOVL7, the cytosol-facing cavity of TMEM120A is surrounded by positively charged residues. An extra density was observed in the cavity, the size and shape of which is reminiscent of the 3′-phoshoadenosine group of acyl-CoA (Fig. 1k; Supplementary Fig. S4b). TMEM120A is unlikely to have the same function as ELOVL7, as another key residue His181 in the catalytic site of ELOVL7 is not conserved in TMEM120A (Fig. 1j). Nevertheless, the similarity of the overall fold, cytosol-facing positively charged pocket, and a potential bound molecule in the cavity imply TMEM120A may be an enzyme in lipid metabolism that catalyzes a different reaction from ELOVL7.

In this study, the almost identical structures between TMEM120A and TMEM120B, and our electrophysiological characterizations do not support the role of TMEM120A as an MSC. The overall structure of TMEM120A revealed in our study is not similar to any previously reported MSC structures. The ion permeation path of most MSCs, including MscL and MscS from bacteria, NOMPC from fly, and TRAAK and Piezo from mammal, are along the symmetric axis. The dimeric TMEM120A is reminiscent of OSCAs from plant, whose ion permeation path is along each protomer. Although TMEM120A shows the ability to permeate ions as measured in bilayer system, our results indicate TMEM120A does not respond to poking or stretch mechanical stimuli in heterologous expression system. We even cannot conclude TMEM120A is an ion channel as conducting currents were also measured for several non-channel proteins in the bilayer system^8^. The structural similarity between TMEM120A and ELOVL7 suggests that TMEM120A may function as an enzyme in lipid metabolism^3^. The exact function of TMEM120A awaits to be further investigated.

## Supplementary information

Supplementary Figures and Table

## Data Availability

Atomic coordinates and corresponding EM maps of the human TMEM120A (PDB: 7CXR; EMDB: EMD-30495) and TMEM120B (PDB: 7F73; EMDB: EMD-31484) have been deposited in the Protein Data Bank (http://www.rcsb.org) and the Electron Microscopy Data Bank (https://www.ebi.ac.uk/pdbe/emdb/), respectively.
